# Association between serum visfatin levels and atherosclerotic plaque in patients with type 2 diabetes

**DOI:** 10.1186/s13098-019-0455-5

**Published:** 2019-07-24

**Authors:** Long-Yi Zheng, Xia Xu, Ren-Hui Wan, Sheng Xia, Jin Lu, Qin Huang

**Affiliations:** 1 Department of Endocrinology, Changhai Hospital, Second Military Medical University, 168 Changhai Road, Shanghai, 200433 China; 2 Department of Rheumatology and Immunology, Changhai Hospital, Second Military Medical University, 168 Changhai Road, Shanghai, 200433 China

**Keywords:** Visfatin, Type 2 diabetes mellitus, Atherosclerosis, Carotid plaques

## Abstract

**Background:**

Visfatin is a multifaceted protein that plays an important role in regulating a variety of physiological and pathological processes, including obesity, diabetes and cardiovascular disease. However, circulating visfatin levels in atherosclerosis plaque progression in patients with type 2 diabetes, or its association with the vascular territory affected remain unclear.

**Methods:**

We evaluated the relationship between visfatin levels and carotid or femoral artery atherosclerosis in Chinese patients with type 2 diabetes. Serum levels of visfatin were measured by enzyme-linked immunosorbent assay (ELISA) in 97 inpatients with type 2 diabetes. Carotid and/or femoral atherosclerotic plaques were detected by B-mode ultrasound.

**Results:**

Serum visfatin levels were elevated in the group with atherosclerotic plaques compared to the control group without plaques [0.68 (0.46–1.58) versus 0.45 (0.23–0.76) ng/mL, respectively, *P *= 0.0002]. Patients with carotid plaques showed higher visfatin levels than those with or without femoral plaques. Pearson’s correlation analysis showed that serum visfatin levels were positively correlated with waist circumference (*r* = 0.226, *P *= 0.029), waist-hip ratio (*r* = 0.221, *P *= 0.032), TG (*r* = 0.222, *P *= 0.030) and number of plaques (*r* = 0.275, *P *= 0.009). Logistic regression analysis showed that a higher serum visfatin level was an independent predictor for the presence of atherosclerotic plaques.

**Conclusions:**

In conclusion, among patients with T2DM, serum visfatin levels were elevated in those with atherosclerotic plaques, especially in patients with carotid atherosclerotic plaques. Serum visfatin may serve as a predictor of atherosclerotic plaques in patients with T2DM.

**Electronic supplementary material:**

The online version of this article (10.1186/s13098-019-0455-5) contains supplementary material, which is available to authorized users.

## Background

Cardiovascular disease is the leading cause of death in China [1, 2]. Atherosclerosis is the underlying cause of cardiovascular disease and inflammation and oxidative stress are thought to play an important role in this complex process [3, 4]. Increasing evidence has highlighted that diabetes is a major risk factor for the development of atherosclerosis. Epidemiologic and experimental data further suggested that the pathohistological characteristics and clinical manifestations of atherosclerosis differed depending on the vascular territory involved [5]. Carotid and femoral atherosclerotic plaques are associated with distinctive therapeutic outcomes [6]. Exploring the roles of different inflammatory mediators within the carotid and femoral plaques in patients with diabetes will help to clarify the exact pathogenesis of atherosclerotic lesions. However, the risk factors and mechanisms contributing to development of these complications are inadequately investigated.

Visfatin, also known as nicotinamide phosphoribosyltransferase (NAMPT), or pre-B cell colony-enhancing factor (PBEF), has been identified as a novel and multifaceted protein, which plays an important role in regulating a variety of physiological and pathological functions [7]. In the context of metabolic diseases, elevated circulating levels of visfatin have been proposed as markers of inflammation and endothelial dysfunction. The association between circulating visfatin with cardiovascular disease has also been extensively analyzed. High visfatin plasma levels may promote vascular inflammation and atherosclerotic plaque destabilization [8–10]. Increased serum visfatin levels have been associated with carotid atherosclerosis in patients with type 2 diabetes or metabolic syndrome [11, 12]. However, circulating visfatin levels in atherosclerosis plaque progression in patients with type 2 diabetes, or its association with the vascular territory affected remain unclear.

The aim of this study was to explore the association between circulating visfatin and carotid, femoral artery atherosclerosis in a Chinese population with type 2 diabetes and to analyze the difference of visfatin levels between patients with carotid and femoral plaques.

## Methods

### Study population and design

This was an observational cross-sectional study of a single-center cohort conducted at the department of endocrinology in Changhai Hospital (Shanghai, China). From June 2017 to December 2017, a total of 97 inpatients with type 2 diabetes were consecutively recruited. The diagnostic criteria of diabetes were based on the American Diabetes Association standards. We excluded patients with type 1 diabetes, acute complications of diabetes such as ketoacidosis and hyperosmolar state, hepatic and renal failure, liver cirrhosis, acute or chronic inflammatory diseases, malignancy, pregnancy or lactation, and autoimmune diseases. The study was approved by the ethics committee of the Changhai Hospital. Written informed consent was obtained before the data collection and analysis.

### Clinical and biochemical assessment

Basic anthropometric measurements of all subjects, including height, body weight, waist circumference, hipline circumference, were collected according to standard protocols. Laboratory data was measured from blood samples, which were taken in the morning after an overnight fast of at least 10 h. Blood cell analysis (including white blood cell (WBC) count, total neutrophils, lymphocytes, and platelet count) was performed on the Sysmex XN-9000 analyzer (Sysmex Co., Kobe, Japan). Plasma glucose, serum alanine aminotransferase (ALT), aspartate aminotransferase (AST), γ-glutamyl transpeptidase (GGT), bilirubin, creatinine, uric acid, lipid profile, including total cholesterol, triglycerides, HDL cholesterol (HDL-c), LDL cholesterol (LDL-c) were measured using HITACHI 7600-120 automatic biochemical analyzer (Hitachi Co., Japan). Endogenous creatinine clearance rate (Ccr) was calculated using the following formula: Urine Cr (mg/dL) × 24 h urine volume (dL)/Serum Cr (mg/dL) × 1440 (min/day). Hemoglobin A1c (HbA1c) was measured by high-pressure liquid chromatography. Fasting blood glucose (FBG), and 2 h postprandial blood glucose (PBG), were measured using the glucose oxidase method. Insulin and C-peptide levels were measured on an automatic analyzer (Roche-E601, Roche Diagnostics, Germany). Insulin resistance status was assessed using the homeostasis model assessment of insulin resistance (HOMA-IR) according to the following formula: fasting serum insulin (mIU/L) × fasting plasma glucose (mmol/L)/22.5.

### Enzyme-linked immunosorbent assay (ELISA)

Serum levels of visfatin were measured using the commercially available enzyme-linked immunosorbent assay (ELISA) kits according to the manufacturer’s instructions (Raybiotech Inc., GA, USA). The intra-assay and inter-assay coefficients of variation were less than 10% in enzyme immunoassays.

### Carotid and femoral ultrasound measurement

B-mode ultrasound of the carotid/femoral arteries was performed using the Philips rE33 Ultrasound System (Philips Healthcare, Andover, MA). A single experienced reader blinded to clinical follow-up data performed the analysis of the presence of atheromatous plaques. We defined plaques as having an IMT ≥ 1.3 mm and/or a focal protrusion into the lumen with a thickness of at least 50% more than the adjacent intima-media complex.

### Statistics

Normally distributed data were expressed as mean ± standard deviation (SD), and data with skewed distribution were shown as medians (Q1–Q3). Differences between groups were evaluated with Student’s t test or Mann–Whitney U test. The association between serum visfatin levels and other variables were evaluated with Spearman’s correlation analysis. Logistic regression analysis was performed to evaluate the odds ratio of Atherosclerotic Plaques. Two-tailed P values < 0.05 were considered to be statistically significant.

The sample size calculations for the comparison between the mean of 2 independent groups were performed using PASS 11 (β = 0.8, α = 0.05, μ1 = 0.57, μ2  = 1.97, s1 = 0.59, s2 = 1.99). According to these calculations, the minimal sample size required for each group was 19 subjects.

## Results

### Demographic and clinical characteristics of the subjects

Overall, our study included 97 patients with T2DM who were admitted to our hospital. The demographic and clinical characteristics of the recorded patients are given in Additional file 1: Table S1. The mean age of the subjects was 54.2 ± 7.2 years. Male subjects had higher levels of waist-hip ratio, white blood cell count, and creatinine compared to female subjects. In contrast, male subjects had significantly lower levels of HDL-c and 2 h postprandial plasma glucose than female subjects. There was no difference in serum visfatin levels between male and female subjects.

### Visfatin concentrations in patients with carotid or/and femoral arteries plaques

According to the findings of the B-mode ultrasonic examination of carotid and femoral arteries, all patients were divided into two groups: plaque group (56 subjects) and no plaque group (41 subjects). As shown in Table 1, age was higher in the group with atherosclerotic plaques. By contrast, serum endogenous creatinine clearance rate (Ccr), serum total bilirubin (STB), direct (conjugated) bilirubin (SDB) and indirect (unconjugated) bilirubin (SIB) were significantly lower in the group with atherosclerotic plaques. After adjusting for age, these differences still exist, with the exception of Ccr. Furthermore, serum visfatin levels were elevated in the group with atherosclerotic plaques (*P *= 0.0002). Moreover, in both male and female subgroup, serum levels of visfatin were significantly higher in patients with atherosclerotic plaques compared to patients without plaques (male subgroup: 0.63(0.43–3.01) versus 0.48(0.27–0.76) ng/mL, *P *= 0.005; female subgroup: 0.71(0.52–0.90) versus 0.39(1.18–0.64) ng/mL, *P *= 0.008). We also analyzed serum visfatin levels according to the type of vascular territory affected (femoral or carotid arteries). Interestingly, as shown in Table 2, patients with carotid plaques showed higher visfatin levels compared to those with femoral plaques or without plaques.

**Table 1:** The demographic and clinical characteristics of the diabetic patients with and without plaques

Variables	Plaques (n = 56)	No plaques (n = 41)	*P*-value
Age (years)	54.04 ± 12.09	47.20 ± 13.88	*0.011*
BMI (kg/m^2^)	27.91 ± 3.21	27.82 ± 4.34	0.913
Diabetes duration (year)	5.86 ± 5.27	5.48 ± 6.06	0.747
Waist circumference (cm)	98.33 ± 8.89	98.82 ± 11.36	0.817
Hipline circumference (cm)	103 (98, 105.5)	99 (95, 105.75)	0.952
Waist-to-hip ratio (WHR)	0.96 ± 0.06	0.96 ± 0.07	0.803
White blood cell (10^9^/L)	6.80 ± 1.61	6.45 ± 1.84	0.319
Neutrophils (10^9^/L)	3.92 ± 1.23	3.70 ± 1.27	0.385
Lymphocytes (10^9^/L)	2.21 ± 0.66	2.09 ± 0.70	0.376
Platelets (10^9^/L)	205.86 ± 55.05	197.10 ± 64.65	0.474
Alanine aminotransferase (U/L)	33.75 ± 22.01	39.98 ± 26.52	0.21
Aspartate aminotransferase (U/L)	24.80 ± 21.04	25.88 ± 14.77	0.78
γ-Glutamyl transpeptidase (U/L)	49.80 ± 35.87	61.72 ± 41.30	0.135
Total cholesterol (mg/dL)	4.91 ± 1.70	5.62 ± 2.58	0.108
Triglycerides (mg/dL)	3.22 ± 3.77	2.89 ± 3.23	0.653
LDL cholesterol (mg/dL)	2.69 ± 1.02	2.98 ± 1.36	0.240
HDL cholesterol (mg/dL)	0.99 ± 0.31	1.07 ± 0.25	0.180
Serum total bilirubin (STB)	10.6 (8.65, 13.45)	12.8 (10.2, 18)	*0.01*
Direct (conjugated) bilirubin (SDB)	3.2 (2.38, 4.25)	3.8 (2.7, 6.1)	*0.048*
Indirect (unconjugated) bilirubin (SIB)	7.3 (6, 9.9)	8.8 (7.2, 12.7)	*0.021*
Uric acid (μmol/L)	0.37 ± 0.12	0.39 ± 0.25	0.557
Creatinine (mg/dL)	74.11 ± 21.20	67.80 ± 20.51	0.146
Endogenous creatinine clearance rate, Ccr	100.31 ± 26.07	115.43 ± 29.16	*0.009*
Glycated hemoglobin A1c (%)	9.44 ± 1.90	9.48 ± 2.78	0.943
Fasting plasma glucose (mmol/L)	9.32 ± 3.67	9.67 ± 3.60	0.642
2 h postprandial plasma glucose (mmol/L)	17.64 ± 4.15	17.53 ± 4.64	0.904
Fasting C-peptide (ng/mL)	2.62 ± 1.10	2.39 ± 1.37	0.378
2-h postprandial C peptide (ng/mL)	5.79 ± 3.23	5.02 ± 2.96	0.238
Fasting insulin (mIU/L)	17.84 ± 27.99	14.04 ± 11.16	0.419
2 h postprandial insulin (mIU/L)	43.05 ± 46.10	45.98 ± 85.87	0.831
HOMA-IR	4.57 (2.84, 7.77)	4.49 (2.23, 6.88)	0.733
Visfatin (ng/mL)	0.68 (0.46, 1.58)	0.45 (0.23, 0.76)	*0.0002*

**Table 2:** Serum visfatin levels [shown as medians and (Q1–Q3)] according to the vascular territory affected

Parameters	Visfatin (ng/mL)
No plaque (n = 41)	0.45 (0.23–0.76)
Carotid plaques (n = 20)	2.37 (0.74–6.27)^a,b,c^
Femoral plaques (n = 20)	0.49 (0.38–0.66)
Both carotid and femoral plaques (n = 16)	0.68 (0.55–1.25)^a,b^

^a^*P *< 0.01 versus No plaque group

^b^*P *< 0.05 versus femoral plaques group

^c^*P *< 0.05 versus both carotid and femoral plaques group (Mann–Whitney U test)

### Correlations between serum visfatin levels and various clinical parameters

To find out the related factors influencing serum visfatin levels, Pearson’s correlation analysis of clinical and biochemical parameters with visfatin were undertaken. As shown in Table 3, in total subjects, serum visfatin levels were positively correlated with waist circumference (*r *= 0.226, *P *= 0.029), waist-hip ratio (*r *= 0.221, *P *= 0.032), TG (*r* = 0.222, *P *= 0.030) and number of plaques (*r* = 0.275, *P *= 0.009).

**Table 3:** Correlation of visfatin with anthropometric and biochemical variables

Parameter	Visfatin	
	*r*	*P*-value
Age (year)	− 0.190	0.063
Diabetes duration (year)	− 0.142	0.167
Body mass index (kg/cm^2^)	0.058	0.570
Waist circumference (cm)	0.226	*0.029*
Waist-to-hip ratio (WHR)	0.221	*0.032*
Fasting plasma glucose (mmol/L)	0.056	0.587
Fasting C-peptide (ng/mL)	− 0.036	0.732
Total cholesterol (mmol/L)	0.098	0.342
Triglyceride (mmol/L)	0.222	*0.030*
High-density lipoprotein cholesterol (mmol/L)	0.031	0.762
Low-density lipoprotein cholesterol (mmol/L)	− 0.149	0.148
Glycated hemoglobin A1c (%)	0.044	0.673
Endogenous creatinine clearance rate, Ccr	0.053	0.604
Serum total bilirubin (STB)	− 0.077	0.459
Direct bilirubin (SDB)	− 0.048	0.638
Serum indirect bilirubin (SIB)	− 0.052	0.611
Number of plaques	0.275	*0.009*

### Serum visfatin as a predictor of atherosclerotic plaques

In order to assess which factors were independent predictors of the presence of atherosclerotic plaques, multivariable logistic regression analysis was performed. Since many covariates (such as obesity, metabolic syndrome) could affect visfatin levels, the relevant confounding variables (sex, age, BMI, BUN, Cr, FBG, PBG, HbA1C, HOMA-IR, TG, TC, HDL-C, LDL-C) were entered in the full regression model. As shown in Table 4, after controlling for potential confounding variables, older age and higher serum visfatin levels were independent predictors for the presence of atherosclerotic plaques.

**Table 4:** Independent factors for plaques by multivariable logistic regression analysis

Parameters	OR (95% CI)	*P*-value
visfatin	3.315 (1.496–7.349)	0.003
age	1.076 (1.031–1.123)	0.001

## Discussion

In the current study, we demonstrated that serum visfatin levels are significantly elevated in type 2 diabetic patients with atherosclerotic plaques compared to those without plaques. Interestingly, the elevation was most obvious in men with atherosclerotic plaques. Moreover, visfatin was higher in patients with T2DM with carotid plaques compared to those with femoral atherosclerotic plaques or without plaques.

Growing evidence suggests that visfatin is expressed in a wide range of cell types (including adipocytes, immune cells, hepatocytes, myoblasts) [13–15] and can exert different actions in a paracrine or endocrine manner. Therefore, visfatin has arisen as a multifaceted molecule that plays an important role in many physiological and pathophysiological processes [7].

Controversial findings on visfatin levels have been reported across many metabolic diseases. Several studies confirmed an increased level of circulating visfatin in obesity, type 2 diabetes, and metabolic syndrome [16]. On the contrary, some authors claimed that circulating visfatin levels were unmodified or even lower compared to healthy controls [17–19]. Fat mass accumulation and persistent chronic low-grade inflammation are key pathophysiological events of atherothrombosis [20, 21]. In the present study, no significant differences were observed in metabolic risk factors (BMI, waist circumference, waist-to-hip ratio, LDL cholesterol, HDL cholesterol, HOMA-IR) between the group with atherosclerotic plaques and without plaques. Intriguingly, the levels of serum bilirubin, which were end metabolic products of heme degradation with potent antioxidant and anti-inflammatory properties, were found to be higher in patients without atherosclerotic plaques. This was in agreement with the previous report by Antonio et al. [22], who stated that high serum bilirubin might protect, in part, dyslipidemic individuals from atherosclerosis.

In the last decade, there is growing interest in the potential role of visfatin in the pathogenesis of metabolic related cardiovascular complications. Increased visfatin serum levels were reported to be correlated with carotid plaque vulnerability in patients with carotid stenosis [23], and visfatin levels can be a predictor of cardiovascular mortality and morbidity in acute ischemic stroke [24]. In agreement with the previous report by Kadoglou et al. [11], we found that serum visfatin concentrations were elevated in patients with carotid plaques. Spearman correlation analysis showed positive correlations between visfatin levels and an unfavorable metabolic profile (high levels of waist circumference, waist-to-hip ratio and TG). We further demonstrated that elevated serum visfatin levels were independent predictors for the presence of atherosclerotic plaques. Collectively, our study supported a role for visfatin as a potential biomarker of metabolic related cardiovascular complications.

Few previous epidemiologic studies have addressed the possible gender differences in visfatin-concentrations. There was no difference in serum visfatin concentration between male subjects and female subjects in the entire cohort. In both male and female subproup, serum levels of visfatin were significantly higher in patients with atherosclerosis compared to patients without atherosclerosis (*P *< 0.01). Therefore, different from another adipose tissue-secreted hormone, leptin, which has confirmed gender-based difference [25], the actions of visfatin may not be regulated by sex hormones. In fact, numerous studies have reported the relationship between visfatin levels and lipoprotein metabolites in patients with metabolic disorders [26–28]. In line with these reports, we also found that serum visfatin levels correlated positively with waist circumference, waist-hip ratio, and TG.

Another important finding of our study is the difference in visfatin concentrations observed depending on the vascular territory affected. Visfatin was higher in patients with T2D with carotid plaques compared with those with femoral atherosclerotic plaques or without plaques. One possible explanation is that the differences in visfatin serum concentration between patients with carotid and femoral plaques could be attributed to differences in the cellular composition of plaques. Compared with femoral plaques, carotid plaques are characterized by a higher inflammatory cell content and lower fibrotic content [29]. In fact, although the overall levels of visfatin did not differ between symptomatic and asymptomatic carotid artery stenosis patients [30], significantly high visfatin levels have been observed in unstable carotid atherosclerotic plaque secretome compared with non-atherosclerotic mammary artery secretome [9].

As a pro-inflammatory cytokine, visfatin can upregulate the production of IL-1β, IL-6 and TNF-α in human monocytes and vascular endothelial cells [31]. Higher visfatin serum concentration may reflect a higher inflammatory state, thereby contributing to the formation of carotid plaques. In addition, in the progression of chronic kidney disease, serum levels of visfatin were found to be closely correlated with IL-6 and common carotid arteries intima-media thickness [32]. In fact, we indeed observed a higher level of circulating IL-6 in patients with a higher visfatin serum concentration (data not shown).

Nevertheless, beyond being just a clinical marker for cardiovascular diseases, visfatin plays an important role in regulating vascular inflammation and atherosclerosis. On the one hand, extracellular visfatin can promote proliferation of human vascular smooth muscle cells through a NAMPT-dependent mechanism [33]. On the other hand, growing scientific evidence supports that visfatin can promote the vascular inflammation through its immunomodulatory properties on different cell types, including vascular smooth muscle cells, endothelial cells and immune cells [34–37]. NAMPT has been reported to increase in inflammatory M1 macrophages compared to in anti-inflammatory M2 macrophages, suggesting that NAMPT could contribute to plaque inflammation in atherosclerotic disorders at least partly through its effect on macrophages [38]. In addition, Alexander et al. have also found that elevated level of visfatin was an independent predictor for declined numbers of non-classical endothelial progenitor cells in T2DM individuals [39]. Therefore, visfatin has emerged as a promising pharmacological target in the context of cardiovascular complications.

Our study has several limitations that need to be discussed. Firstly, the study was based on data obtained cross-sectionally with a relatively small sample size of Chinese T2DM patients at a single center. Prospective studies in larger samples and in other ethnic groups are needed to determine the role of serum visfatin in atherosclerotic plaque in patients with type 2 diabetes. Secondly, single measurements of serum visfatin level may underestimate the strength of the associations in cohorts. Furthermore, most patients with diabetes were kept on their current hypoglycaemic medications, which could affect the potential relationships under investigation. Finally, atherosclerosis is a slow, progressive disease with a complex pathological process, therefore a single biomarker would not be sufficient to detect or predict atherosclerotic plaques. Serum visfatin levels could provide additional information about the risk factors of developing cardiovascular disease.

## Conclusions

Our study demonstrated that among patients with T2DM, serum visfatin levels were elevated in those with atherosclerotic plaques, and especially those with carotid atherosclerotic plaques. Serum visfatin may serve as a predictor of atherosclerotic plaques in patients with T2DM.

## Additional file

**Additional file 1.** The demographic and clinical characteristics of the recorded patients.

## Data Availability

The datasets used and/or analysed during the current study are available from the corresponding author on reasonable request.

## Abbreviations

T2DM: type 2 diabetes mellitus
NAMPT: nicotinamide phosphoribosyltransferase
BMI: body mass index
WC: waist circumference
WHR: waist-to-hip Ratio
ALT: alanine aminotransferase
AST: aspartate aminotransferase
TG: triglycerides
LDL: low-density lipoprotein
HDL: high-density lipoprotein
HOMA-IR: homeostasis-model assessment of insulin resistance

## References

[1] Barkoudah E, Skali H, Uno H, Solomon SD, Pfeffer MA (2012). Mortality rates in trials of subjects with type 2 diabetes. J Am Heart Assoc..

[2] Moran A, Gu D, Zhao D, Coxson P, Wang YC, Chen CS (2010). Future cardiovascular disease in china: markov model and risk factor scenario projections from the coronary heart disease policy model-china. Circ Cardiovasc Qual Outcomes..

[3] Libby P, Ridker PM, Maseri A (2002). Inflammation and atherosclerosis. Circulation.

[4] Gray SP, Di Marco E, Okabe J, Szyndralewiez C, Heitz F, Montezano AC (2013). NADPH oxidase 1 plays a key role in diabetes mellitus-accelerated atherosclerosis. Circulation.

[5] Poredos P, Jezovnik MK (2018). Structure of atherosclerotic plaques in different vascular territories: clinical relevance. Curr Vasc Pharmacol.

[6] Zhou W, Chai H, Ding R, Lam HY (2010). Distribution of inflammatory mediators in carotid and femoral plaques. J Am Coll Surg.

[7] Carbone F, Liberale L, Bonaventura A, Vecchie A, Casula M, Cea M (2017). Regulation and function of extracellular nicotinamide phosphoribosyltransferase/visfatin. Compr Physiol..

[8] Yu PL, Wang C, Li W, Zhang FX (2019). Visfatin level and the risk of hypertension and cerebrovascular accident: a systematic review and meta-analysis. Horm Metab Res.

[9] Auguet T, Aragones G, Guiu-Jurado E, Berlanga A, Curriu M, Martinez S (2016). Adipo/cytokines in atherosclerotic secretomes: increased visfatin levels in unstable carotid plaque. BMC Cardiovasc Disord..

[10] Li B, Zhao Y, Liu H, Meng B, Wang J, Qi T (2016). Visfatin destabilizes atherosclerotic plaques in apolipoprotein E-deficient mice. PLoS ONE.

[11] Kadoglou NP, Sailer N, Moumtzouoglou A, Kapelouzou A, Tsanikidis H, Vitta I (2010). Visfatin (NAMPT) and ghrelin as novel markers of carotid atherosclerosis in patients with type 2 diabetes. Exp Clin Endocrinol Diabetes.

[12] Zhong M, Tan HW, Gong HP, Wang SF, Zhang Y, Zhang W (2008). Increased serum visfatin in patients with metabolic syndrome and carotid atherosclerosis. Clin Endocrinol (Oxf).

[13] Stephens JM, Vidal-Puig AJ (2006). An update on visfatin/pre-B cell colony-enhancing factor, an ubiquitously expressed, illusive cytokine that is regulated in obesity. Curr Opin Lipidol.

[14] Costford SR, Bajpeyi S, Pasarica M, Albarado DC, Thomas SC, Xie H (2010). Skeletal muscle NAMPT is induced by exercise in humans. Am J Physiol Endocrinol Metab.

[15] Garten A, Petzold S, Barnikol-Oettler A, Korner A, Thasler WE, Kratzsch J (2010). Nicotinamide phosphoribosyltransferase (NAMPT/PBEF/visfatin) is constitutively released from human hepatocytes. Biochem Biophys Res Commun.

[16] Romacho T, Sanchez-Ferrer CF, Peiro C (2013). Visfatin/Nampt: an adipokine with cardiovascular impact. Mediators Inflamm.

[17] Saboori S, Hosseinzadeh-Attar MJ, Yousefi Rad E, Hosseini M, Mirzaei K, Ahmadivand Z (2015). The comparison of serum vaspin and visfatin concentrations in obese and normal weight women. Diabetes Metab Syndr..

[18] Rezvan N, Hosseinzadeh-Attar MJ, Masoudkabir F, Moini A, Janani L, Mazaherioun M (2012). Serum visfatin concentrations in gestational diabetes mellitus and normal pregnancy. Arch Gynecol Obstet.

[19] Robinson C, Tsang L, Solomon A, Woodiwiss AJ, Gunter S, Mer M (2018). Nesfatin-1 and visfatin expression is associated with reduced atherosclerotic disease risk in patients with rheumatoid arthritis. Peptides.

[20] Chistiakov DA, Grechko AV, Myasoedova VA, Melnichenko AA, Orekhov AN (2017). Impact of the cardiovascular system-associated adipose tissue on atherosclerotic pathology. Atherosclerosis..

[21] Shi C, Men L, Yu C, Yao J, Bai R, Yang Y (2017). Atherosclerosis associated with dynamic inflammation changes after multifactorial intervention in short-duration type 2 diabetes: a randomized, controlled, 10-year follow-up trial. J Diabetes Complications.

[22] Amor AJ, Ortega E, Perea V, Cofan M, Sala-Vila A, Nunez I (2017). Relationship between total serum bilirubin levels and carotid and femoral atherosclerosis in familial dyslipidemia. Arterioscler Thromb Vasc Biol.

[23] Kadoglou NP, Sailer N, Moumtzouoglou A, Kapelouzou A, Gerasimidis T, Kostakis A (2012). Adipokines: a novel link between adiposity and carotid plaque vulnerability. Eur J Clin Invest.

[24] Kadoglou NP, Fotiadis G, Lambadiari V, Maratou E, Dimitriadis G, Liapis CD (2014). Serum levels of novel adipokines in patients with acute ischemic stroke: potential contribution to diagnosis and prognosis. Peptides.

[25] Couillard C, Mauriege P, Prud’homme D, Nadeau A, Tremblay A, Bouchard C (1997). Plasma leptin concentrations: gender differences and associations with metabolic risk factors for cardiovascular disease. Diabetologia.

[26] Kim JJ, Choi YM, Hong MA, Kim MJ, Chae SJ, Kim SM (2018). Serum visfatin levels in non-obese women with polycystic ovary syndrome and matched controls. Obstet Gynecol Sci..

[27] Liang Z, Wu Y, Xu J, Fang Q, Chen D (2016). Correlations of serum visfatin and metabolisms of glucose and lipid in women with gestational diabetes mellitus. J Diabetes Investig..

[28] Jin H, Jiang B, Tang J, Lu W, Wang W, Zhou L (2008). Serum visfatin concentrations in obese adolescents and its correlation with age and high-density lipoprotein cholesterol. Diabetes Res Clin Pract.

[29] Herisson F, Heymann MF, Chetiveaux M, Charrier C, Battaglia S, Pilet P (2011). Carotid and femoral atherosclerotic plaques show different morphology. Atherosclerosis..

[30] Musialek P, Tracz W, Tekieli L, Pieniazek P, Kablak-Ziembicka A, Przewlocki T (2013). Multimarker approach in discriminating patients with symptomatic and asymptomatic atherosclerotic carotid artery stenosis. J Clin Neurol..

[31] Tilg H, Moschen AR (2008). Role of adiponectin and PBEF/visfatin as regulators of inflammation: involvement in obesity-associated diseases. Clin Sci..

[32] Tang X, Chen M, Zhang W (2013). Association between elevated visfatin and carotid atherosclerosis in patients with chronic kidney disease. Zhong Nan Da Xue Xue Bao Yi Xue Ban..

[33] Wang P, Xu TY, Guan YF, Su DF, Fan GR, Miao CY (2009). Perivascular adipose tissue-derived visfatin is a vascular smooth muscle cell growth factor: role of nicotinamide mononucleotide. Cardiovasc Res.

[34] Romacho T, Azcutia V, Vazquez-Bella M, Matesanz N, Cercas E, Nevado J (2009). Extracellular PBEF/NAMPT/visfatin activates pro-inflammatory signalling in human vascular smooth muscle cells through nicotinamide phosphoribosyltransferase activity. Diabetologia.

[35] Wang P, Vanhoutte PM, Miao CY (2012). Visfatin and cardio-cerebro-vascular disease. J Cardiovasc Pharmacol.

[36] Venter G, Oerlemans FT, Willemse M, Wijers M, Fransen JA, Wieringa B (2014). NAMPT-mediated salvage synthesis of NAD+ controls morphofunctional changes of macrophages. PLoS ONE.

[37] Presumey J, Courties G, Louis-Plence P, Escriou V, Scherman D, Pers YM (2013). Nicotinamide phosphoribosyltransferase/visfatin expression by inflammatory monocytes mediates arthritis pathogenesis. Ann Rheum Dis.

[38] Halvorsen B, Espeland MZ, Andersen GO, Yndestad A, Sagen EL, Rashidi A (2015). Increased expression of NAMPT in PBMC from patients with acute coronary syndrome and in inflammatory M1 macrophages. Atherosclerosis..

[39] Berezin AE, Samura TA, Kremzer AA, Berezina TA, Martovitskaya YV, Gromenko EA (2016). An association of serum vistafin level and number of circulating endothelial progenitor cells in type 2 diabetes mellitus patients. Diabetes Metab Syndr..

