# Supplementary material for: Association between serum visfatin levels and atherosclerotic plaque in patients with type 2 diabetes
Source: Diabetol Metab Syndr. 2019 Jul 24;11:60. doi: 10.1186/s13098-019-0455-5 (PMC6657107; doi:10.1186/s13098-019-0455-5)
Supplement: Supplementary file 1 — Additional file 1. The demographic and clinical characteristics of the recorded patients. [file 13098_2019_455_MOESM1_ESM.docx]

**Additional file 1: Table S1. The demographic and clinical characteristics of the recorded patients.**

| **Variables** | **Men(n=68)** | **Women(n=29)** | ***P* -value** |
| --- | --- | --- | --- |
| Age (years) | 49.51±12.25 | 54.97±14.88 | 0.063 |
| BMI (kg/m2) | 27.86±3.56 | 27.89±4.10 | 0.967 |
| Diabetes duration(year) | 5.46±5.62 | 6.26±5.58 | 0.519 |
| Waist circumference (cm) | 99.48±9.88 | 96.41±9.84 | 0.168 |
| Hipline circumference (cm) | 101.53±7.29 | 104.34±9.10 | 0.150 |
| Waist-to-Hip Ratio (WHR) | 0.98±0.05 | 0.92±0.07 | **<0.001** |
| White blood cell (10^9/L) | 6.88±1.69 | 6.12±1.68 | **0.044** |
| Neutrophils (10^9/L) | 3.96±1.27 | 3.52±1.13 | 0.112 |
| Lymphocytes (10^9/L) | 2.21±0.68 | 2.03±0.66 | 0.228 |
| Platelets (10^9/L) | 199.65±62.14 | 208.03±51.95 | 0.525 |
| Alanine aminotransferase(U/L) | 35.79±24.08 | 37.76±24.48 | 0.715 |
| Aspartate aminotransferase(U/L) | 19.5(15,25.25) | 25(18,36) | 0.074 |
| γ-glutamyl transpeptidase(U/L) | 58.85±41.30 | 44.86±28.84 | 0.106 |
| Total cholesterol (mg/ dl) | 5.20±2.38 | 5.24±1.47 | 0.925 |
| Triglycerides (mg/ dl) | 3.36±3.83 | 2.43±2.67 | 0.238 |
| LDL cholesterol (mg/ dl) | 2.74±1.23 | 2.98±1.05 | 0.381 |
| HDL cholesterol (mg/ dl) | 0.95(0.82,1.07) | 1.13(0.91,1.29) | **0.003** |
| serum total bilirubin (STB) | 13.73±6.54 | 11.28±5.00 | 0.074 |
| direct (conjugated) bilirubin (SDB) | 4.34±2.85 | 3.42±1.69 | 0.108 |
| indirect (unconjugated) bilirubin (SIB) | 9.62±4.49 | 7.86±3.56 | 0.064 |
| uric acid (μmol/L) | 0.32±0.06 | 0.40±0.24 | 0.071 |
| Creatinine (mg/dL) | 77.90±19.74 | 56.31±15.70 | **<0.001** |
| [endogenous creatinine clearance rate , Ccr](http://www.baidu.com/link?url=a-k88uqIQWN7Y0FHYmYOD4naFQttiXUfFuVs5Gdo8u2eAH7p2bgiFEjSZ9p9QBh9uslFpJ7QjdfGdAM2xuC7x_) | 104.42±26.63 | 112.04±31.69 | 0.226 |
| Glycated hemoglobin A1c(%) | 9.40±2.55 | 9.60±1.61 | 0.701 |
| Fasting plasma glucose(mmol/L) | 9.46±4.05 | 9.48±2.38 | 0.981 |
| 2 h postprandial plasma glucose(mmol/L) | 16.87±4.30 | 19.31±4.01 | **0.011** |
| Fasting C-peptide(ng/ml) | 2.56±1.22 | 2.45±1.23 | 0.699 |
| 2-h postprandial C peptide(ng/ml) | 5.37±3.16 | 5.68±3.08 | 0.662 |
| Fasting insulin (mIU/L) | 10.4(6.55,16.18) | 13(7.42,17.9) | 0.256 |
| 2 h postprandial insulin (mIU/L) | 41.05±69.65 | 52.44±53.70 | 0.447 |
| HOMA-IR | 5.73(2.19,8.29) | 4.2(2.77,6.77) | 0.330 |
| Visfatin (ng/ml) | 0.52(0.37,1.27) | 0.63(0.39,0.81) | 0.762 |
